# Incidence and Trends of Infection with Pathogens Transmitted Commonly Through Food — Foodborne Diseases Active Surveillance Network, 10 U.S. Sites, 2006–2013

**Published:** 2014-04-18

**Authors:** Stacy M. Crim, Martha Iwamoto, Jennifer Y. Huang, Patricia M. Griffin, Debra Gilliss, Alicia B. Cronquist, Matthew Cartter, Melissa Tobin-D’Angelo, David Blythe, Kirk Smith, Sarah Lathrop, Shelley Zansky, Paul R. Cieslak, John Dunn, Kristin G. Holt, Susan Lance, Robert Tauxe, Olga L. Henao

**Affiliations:** 1 Division of Foodborne, Waterborne, and Environmental Diseases, National Center for Emerging and Zoonotic Infectious Diseases, CDC; 2 California Department of Public Health; 3 Colorado Department of Public Health and Environment; 4 Connecticut Department of Public Health; 5 Georgia Department of Public Health; 6 Maryland Department of Health and Mental Hygiene; 7 Minnesota Department of Health; 8 University of New Mexico; 9 New York State Department of Health; 10 Oregon Health Authority; 11 Tennessee Department of Health; 12 Food Safety and Inspection Service, US Department of Agriculture; 13 Center for Food Safety and Applied Nutrition, Food and Drug Administration

Foodborne disease continues to be an important problem in the United States. Most illnesses are preventable. To evaluate progress toward prevention, the Foodborne Diseases Active Surveillance Network* (FoodNet) monitors the incidence of laboratory-confirmed infections caused by nine pathogens transmitted commonly through food in 10 U.S. sites, covering approximately 15% of the U.S. population. This report summarizes preliminary 2013 data and describes trends since 2006. In 2013, a total of 19,056 infections, 4,200 hospitalizations, and 80 deaths were reported. For most infections, incidence was well above national *Healthy People 2020* incidence targets and highest among children aged <5 years. Compared with 2010–2012, the estimated incidence of infection in 2013 was lower for *Salmonella*, higher for *Vibrio*, and unchanged overall.† Since 2006–2008, the overall incidence has not changed significantly. More needs to be done. Reducing these infections requires actions targeted to sources and pathogens, such as continued use of *Salmonella* poultry performance standards and actions mandated by the Food Safety Modernization Act (FSMA) (1). FoodNet provides federal and state public health and regulatory agencies as well as the food industry with important information needed to determine if regulations, guidelines, and safety practices applied across the farm-to-table continuum are working.

FoodNet conducts active, population-based surveillance for laboratory-confirmed infections caused by *Campylobacter*, *Cryptosporidium*, *Cyclospora*, *Listeria*, *Salmonella*, Shiga toxin–producing *Escherichia coli* (STEC) O157 and non-O157, *Shigella*, *Vibrio*, and *Yersinia* in 10 sites covering approximately 15% of the U.S. population (an estimated 48 million persons in 2012).§ FoodNet is a collaboration among CDC, 10 state health departments, the U.S. Department of Agriculture’s Food Safety and Inspection Service (USDA-FSIS), and the Food and Drug Administration (FDA). Hospitalizations occurring within 7 days of specimen collection are recorded, as is the patient’s vital status at hospital discharge, or at 7 days after specimen collection if the patient was not hospitalized. Hospitalizations and deaths that occur within 7 days are attributed to the infection. Surveillance for physician-diagnosed postdiarrheal hemolytic uremic syndrome (HUS), a complication of STEC infection characterized by renal failure, is conducted through a network of nephrologists and infection preventionists and by hospital discharge data review. This report includes 2012 HUS data for persons aged <18 years.

Incidence was calculated by dividing the number of laboratory-confirmed infections in 2013 by U.S. Census estimates of the surveillance area population for 2012.¶ Incidence of culture-confirmed bacterial infections and laboratory-confirmed parasitic infections (e.g., identified by enzyme immunoassay) are reported. A negative binomial model with 95% confidence intervals (CIs) was used to estimate changes in incidence from 2010–2012 to 2013 and from 2006–2008 to 2013 (2). Change in the overall incidence of infection with six key foodborne pathogens was estimated (3). For STEC non-O157, only change since 2010–2012 was assessed because diagnostic practices changed before then; for *Cyclospora*, change was not assessed because data were sparse. For HUS, incidence was compared with 2006–2008. The number of reports of positive culture-independent diagnostic tests (CIDTs) without corresponding culture confirmation is included for *Campylobacter*, *Listeria*, *Salmonella*, *Shigella*, STEC, *Vibrio*, and *Yersinia*.

## Cases of Infection, Incidence, and Trends

In 2013, FoodNet identified 19,056 cases of infection, 4,200 hospitalizations, and 80 deaths (Table). The number and incidence per 100,000 population were *Salmonella* (7,277 [15.19]), *Campylobacter* (6,621 [13.82]), *Shigella* (2,309 [4.82]), *Cryptosporidium* (1,186 [2.48]), STEC non-O157 (561 [1.17]), STEC O157 (552 [1.15]), *Vibrio* (242 [0.51]), *Yersinia* (171 [0.36]), *Listeria* (123 [0.26]), and *Cyclospora* (14 [0.03]). Incidence was highest among persons aged ≥65 years for *Cyclospora*, *Listeria*, and *Vibrio* and among children aged <5 years for all the other pathogens.

Among 6,520 (90%) serotyped *Salmonella* isolates, the top serotypes were Enteritidis, 1,237 (19%); Typhimurium, 917 (14%); and Newport, 674 (10%). Among 231 (95%) speciated *Vibrio* isolates, 144 (62%) were *V. parahaemolyticus*, 27 (12%) were *V. alginolyticus*, and 21 (9%) were *V. vulnificus*. Among 458 (82%) serogrouped STEC non-O157 isolates, the top serogroups were O26 (34%), O103 (25%), and O111 (14%).

**FIGURE 1:**
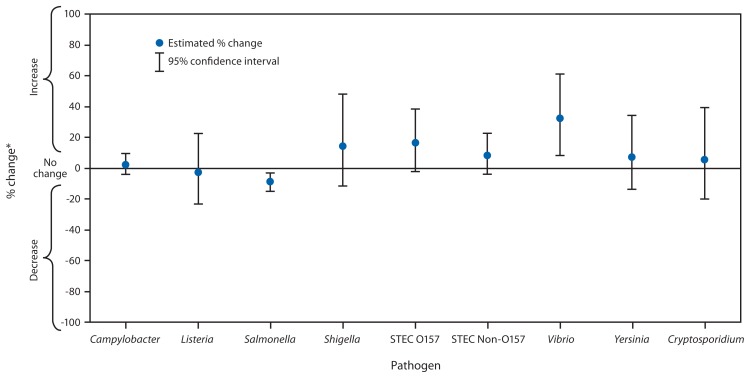
Estimated percentage change in incidence of culture-confirmed bacterial and laboratory-confirmed parasitic infections in 2013 compared with average annual incidence during 2010–2012, by pathogen — Foodborne Diseases Active Surveillance Network, United States **Abbreviations:** CI = confidence interval; STEC = Shiga toxin–producing *Escherichia coli*. * No significant change = 95% CI is both above and below the no change line; significant increase = estimate and entire CI are above the no change line; significant decrease = estimate and entire CI are below the no change line.

**FIGURE 2:**
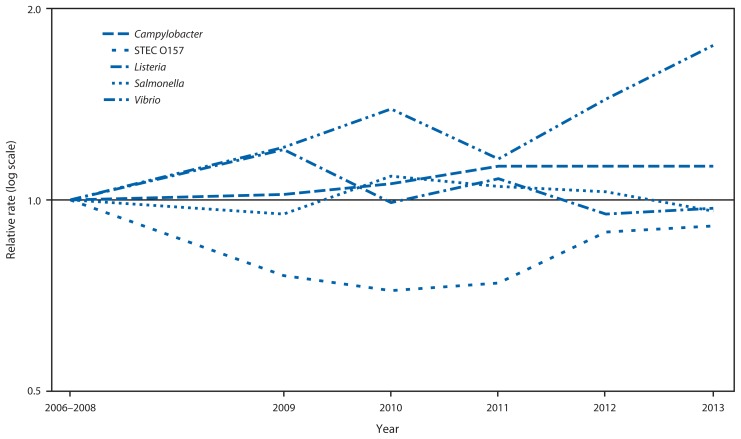
Relative rates of culture-confirmed infections with *Campylobacter*, STEC* O157, *Listeria*, *Salmonella*, and *Vibrio* compared with 2006–2008 rates, by year — Foodborne Diseases Active Surveillance Network, United States, 2006–2013^†^ * Shiga toxin–producing *Escherichia coli*. ^†^ The position of each line indicates the relative change in the incidence of that pathogen compared with 2006–2008. The actual incidences of these infections cannot be determined from this figure.

Compared with 2010–2012, the 2013 incidence was significantly lower for *Salmonella* (9% decrease; CI = 3%–15%), higher for *Vibrio* (32% increase; CI = 8%–61%) and not significantly changed for other pathogens (Figure 1). Compared with 2006–2008, the 2013 incidence was significantly higher for *Campylobacter* and *Vibrio* (Figure 2). The overall incidence of infection with six key foodborne pathogens was not significantly different in 2013 compared with 2010–2012 or 2006–2008.

Compared with 2010–2012, the 2013 incidence of infection with specific *Salmonella* serotypes was significantly lower for Enteritidis (14% decrease; CI = 0.2%–25%) and Newport (32% decrease; CI = 17%–44%) and not significantly changed for Typhimurium. Compared with 2006–2008, however, the 2013 incidence of infection was significantly changed only for Typhimurium (20% decrease; CI = 10%–28%).

Among 62 cases of postdiarrheal HUS in children aged <18 years (0.56 cases per 100,000) in 2012, 38 (61%) occurred in children aged <5 years (1.27 cases per 100,000). Compared with 2006–2008, the incidence was significantly lower for children aged <5 years (36% decrease; CI = 9%–55%) and for children aged <18 years (31% decrease; CI = 7%–49%).

In addition to culture-confirmed infections (some with a positive CIDT result), there were 1,487 reports of positive CIDTs that were not confirmed by culture, either because the specimen was not cultured at either the clinical or public health laboratory or because a culture did not yield the pathogen. For 1,017 *Campylobacter* reports in this category, 430 (42%) had no culture, and 587 (58%) were culture-negative. For 247 STEC reports, 59 (24%) had no culture, and 188 (76%) were culture-negative. The Shiga toxin–positive result was confirmed for 65 (34%) of 192 broths sent to a public health laboratory. The other reports of positive CIDT tests not confirmed by culture were of *Shigella* (147), *Salmonella* (69), *Vibrio* (four), *Listeria* (two), and *Yersinia* (one).

### Discussion

The incidence of laboratory-confirmed *Salmonella* infections was lower in 2013 than 2010–2012, whereas the incidence of *Vibrio* infections increased. No changes were observed for infection with *Campylobacter*, *Listeria*, STEC O157, or *Yersinia*, the other pathogens transmitted commonly through food for which *Healthy People 2020* targets exist. The lack of recent progress toward these targets points to gaps in the current food safety system and the need for more food safety interventions.

Although the incidence of *Salmonella* infection in 2013 was lower than during 2010–2012, it was similar to 2006–2008, well above the national *Healthy People* target. *Salmonella* organisms live in the intestines of many animals and can be transmitted to humans through contaminated food or water or through direct contact with animals or their environments; different serotypes can have different reservoirs and sources. Enteritidis, the most commonly isolated serotype, is often associated with eggs and poultry. The incidence of Enteritidis infection was lower in 2013 compared with 2010–2012, but not compared with 2006–2008. This might be partly explained by the large Enteritidis outbreak linked to eggs in 2010.** Ongoing efforts to reduce contamination of eggs include FDA’s Egg Safety Rule, which requires shell egg producers to implement controls to prevent contamination of eggs on the farm and during storage and transportation.†† FDA required compliance by all egg producers with ≥50,000 laying hens by 2010 and by producers with ≥3,000 hens by 2012. Reduction in Enteritidis infection has been one of five high-priority goals for the U.S. Department of Health and Human Services since 2012.§§

In 2013, the incidence of *Vibrio* infections was the highest observed in FoodNet to date, though still much lower than that of *Salmonella* or *Campylobacter*. *Vibrio* infections are most common during warmer months, when waters contain more *Vibrio* organisms. Many infections follow contact with seawater (4), but about 50% of domestically acquired infections are transmitted through food, most commonly oysters (5). Foodborne infections can be prevented by postharvest treatment of oysters with heat, freezing, or high pressure, by thorough cooking, or by not eating oysters during warmer months (6). During the summers of 2012 and 2013, many *V. parahaemolyticus* infections of a strain previously traced only to the Pacific Northwest were associated with consumption of oysters and other shellfish from several Atlantic Coast harvest areas.¶¶ *V. alginolyticus*, the second most common *Vibrio* reported to FoodNet in 2013, typically causes wound and soft-tissue infections among persons who have contact with water (7).

The continued decrease in the incidence of postdiarrheal HUS has not been matched by a decline in STEC O157 infections. Possible explanations include unrecognized changes in surveillance, improvements in management of STEC O157 diarrhea, or an actual decrease in infections with the most virulent strains of STEC O157. It is possible that more stool specimens are being tested for STEC, resulting in increased detection of milder infections than in the past. Continued surveillance is needed to determine if this pattern holds.

CIDTs are increasingly used by clinical laboratories to diagnose bacterial enteric infections, a trend that will challenge the ability to identify cases, monitor trends, detect outbreaks, and characterize pathogens (8). Therefore, FoodNet began tracking CIDT-positive reports and surveying clinical laboratories about their diagnostic practices. The adoption of CIDTs has varied by pathogen and has been highest for STEC and *Campylobacter*. Positive CIDTs frequently cannot be confirmed by culture, and the positive predictive value varies by the CIDT used. For STEC, most specimens identified as Shiga toxin–positive were sent to a public health laboratory for confirmation. However, for other pathogens the fraction of specimens from patients with a positive CIDT sent for confirmation likely is low because no national guidelines regarding confirmation of CIDT results currently exist. As the number of approved CIDTs increases, their use likely will increase rapidly. Clinicians, clinical and public health laboratorians, public health practitioners, regulatory agencies, and industry must work together to maintain strong surveillance to detect dispersed outbreaks, measure the impact of prevention measures, and identify emerging threats.

The findings in this report are subject to at least five limitations. First, health-care–seeking behaviors and other characteristics of the population in the surveillance area might affect the generalizability of the findings. Second, some agents transmitted commonly through food (e.g., norovirus) are not monitored by FoodNet because clinical laboratories do not routinely test for them. Third, the proportion of illnesses transmitted by nonfood routes differs by pathogen; data provided in this report are not limited to infections from food. Fourth, in some fatal cases, infection with the enteric pathogen might not have been the primary cause of death. Finally, changes in incidence between periods can reflect year-to-year variation during those periods rather than sustained trends.

Most foodborne illnesses can be prevented, and progress has been made in decreasing contamination of some foods and reducing illness caused by some pathogens since 1996, when FoodNet began. More can be done; surveillance data provide information on where to target prevention efforts. In 2011, USDA-FSIS tightened its performance standard for *Salmonella* contamination of whole broiler chickens; in 2013, 3.9% of samples tested positive (Christopher Aston, USDA-FSIS, Office of Data Integration and Food Protection; personal communication; 2014). Because most chicken is purchased as cut-up parts, USDA-FSIS conducted a nationwide survey of raw chicken parts in 2012 and calculated an estimated 24% prevalence of *Salmonella* (9). In 2013, USDA-FSIS released its *Salmonella Action Plan* that indicates that USDA-FSIS will conduct a risk assessment and develop performance standards for poultry parts during 2014, among other key activities (10). The Food Safety Modernization Act of 2011 gives FDA additional authority to regulate food facilities, establish standards for safe produce, recall contaminated foods, and oversee imported foods; it also calls on CDC to strengthen surveillance and outbreak response (1). For consumers, advice on safely buying, preparing, and storing foods prone to contamination is available online.

What is already known on this topic?

The incidences of infection caused by *Campylobacter*, *Salmonella*, Shiga toxin–producing *Escherichia coli* O157, and *Vibrio* are well above their respective *Healthy People 2020* targets. Foodborne illness continues to be an important public health problem.

What is added by this report?

In 2013, a total of 19,056 infections, 4,200 hospitalizations, and 80 deaths were reported to the Foodborne Diseases Active Surveillance Network (FoodNet). For most infections, incidence was highest among children aged <5 years. In 2013, compared with 2010–2012, the estimated incidence of infection was unchanged overall, lower for *Salmonella*, and higher for *Vibrio* infections, which have been increasing in frequency for many years. The number of patients being diagnosed by culture-independent diagnostic tests (CIDT) is increasing.

What are the implications for public health practice?

Reducing the incidence of foodborne infections requires greater commitment and more action to implement measures to reduce contamination of food. Monitoring the incidence of these infections is becoming more difficult because some laboratories are now using CIDTs, and some do not follow up a positive CIDT result with a culture.

## Figures and Tables

**TABLE:** Number of cases of culture-confirmed bacterial and laboratory-confirmed parasitic infection, hospitalizations, and deaths, by pathogen — Foodborne Diseases Active Surveillance Network, United States, 2013*

	Cases			Hospitalizations		Deaths	
			
Pathogen	No.	Incidence†	Objective§	No.	(%)	No.	(%)
**Bacteria**							
*Campylobacter*	6,621	13.82	8.5	1,010	(15)	12	(0.2)
*Listeria*	123	0.26	0.2	112	(91)	24	(19.5)
*Salmonella*	7,277	15.19	11.4	2,003	(28)	27	(0.4)
*Shigella*	2,309	4.82	N/A¶	450	(19)	3	(0.1)
STEC O157	552	1.15	0.6	210	(38)	2	(0.4)
STEC non-O157	561	1.17	N/A	76	(14)	2	(0.4)
*Vibrio*	242	0.51	0.2	55	(23)	2	(0.8)
*Yersinia*	171	0.36	0.3	55	(32)	4	(2.3)
**Parasites**							
*Cryptosporidium*	1,186	2.48	N/A	227	(19)	4	(0.3)
*Cyclospora*	14	0.03	N/A	2	(14)	0	(0.0)
**Total**	**19,056**			**4,200**		**80**	

**Abbreviations:** N/A = not available; STEC = Shiga toxin–producing *Escherichia coli*.

* Data for 2013 are preliminary.

† Per 100,000 population.

§ *Healthy People 2020* objective targets for incidence of *Campylobacter*, *Listeria*, *Salmonella*, STEC O157, *Vibrio*, and *Yersinia* infections per 100,000 population.

¶ No national health objective exists for these pathogens.

## Footnotes

* Additional information available at http://www.cdc.gov/foodnet.

† The overall incidence of infection combines data for *Campylobacter*, *Listeria*, *Salmonella*, STEC O157, *Vibrio*, and *Yersinia*, six key bacterial pathogens for which >50% of illnesses are estimated to be transmitted by food.

§ FoodNet personnel regularly contact clinical laboratories to ascertain all laboratory-confirmed infections in residents of the surveillance areas.

¶ Final incidence rates will be reported when population estimates for 2013 are available.

** Additional information available at http://www.cdc.gov/salmonella/enteritidis/index.html.

†† Additional information available at http://www.fda.gov/food/guidanceregulation/guidancedocumentsregulatoryinformation/eggs/ucm170615.htm.

§§ Additional information available at http://www.hhs.gov/strategic-plan/appendixb3.html.

¶¶ Additional information available at http://www.cdc.gov/vibrio/investigations/index.html.

## References

[1] Food and Drug Administration (2011). FDA Food Safety Modernization Act.

[2] Henao OL, Scallan E, Mahon B, Hoekstra RM (2010). Methods for monitoring trends in the incidence of foodborne diseases: Foodborne Diseases Active Surveillance Network 1996–2008. Foodborne Pathog Dis.

[3] Henao OL, Crim SM, Hoekstra RM (2012). Calculating a measure of overall change in the incidence of selected laboratory-confirmed infections with pathogens transmitted commonly through food, Foodborne Diseases Active Surveillance Network (FoodNet), 1996–2010. Clin Infect Dis.

[4] Shapiro RL, Altekruse S, Hutwagner L (1998). The role of Gulf Coast oysters harvested in warmer months in *Vibrio vulnificus* infections in the United States, 1988–1996. J Infect Dis.

[5] CDC (2013). National enteric disease surveillance: COVIS annual summary, 2011.

[6] Vugia DJ, Tabnak F, Newton AE (2013). Impact of 2003 state regulation on raw oyster-associated *Vibrio vulnificus* illnesses and deaths, California, USA. Emerg Infect Dis.

[7] Dechet AM, Yu PA, Koram N, Painter J (2008). Nonfoodborne *Vibrio* infections: an important cause of morbidity and mortality in the United States, 1997–2006. Clin Infect Dis.

[8] Cronquist AB, Mody RK, Atkinson R (2012). Impacts of culture-independent diagnostic practices on public health surveillance for bacterial enteric pathogens. Clin Infect Dis.

[9] US Department of Agriculture, Food Safety and Inspection Service (2013). The Nationwide Microbiological Baseline Data Collection Program: Raw Chicken Parts Survey, January 2012–August 2012.

[10] US Department of Agriculture, Food Safety and Inspection Service (2013). Strategic Performance Working Group *Salmonella* action plan.

